# PReS-FINAL-2336: Induction of MDSCS in Muckle-Wells syndrome

**DOI:** 10.1186/1546-0096-11-S2-P326

**Published:** 2013-12-05

**Authors:** N Rieber, A Brand, D Neri, T Hall, I Schäfer, S Hansmann, J Kümmerle-Deschner, D Hartl

**Affiliations:** 1 Children's Hospital, Autoinflammation Reference Center, University of Tübingen, Tübingen, Germany

## Introduction

Muckle-Wells syndrome (MWS) is caused by mutations in the *NLRP3*-gene encoding cryopyrin, leading to overproduction of IL-1β and other NLRP3 inflammasome products. Myeloid-derived suppressor cells (MDSCs) represent a novel innate immune cell subset, are generated in tumor, infective, and proinflammatory microenvironments and are capable of suppressing T cell responses. Consequently, MDSCs are considered a key intermediary in balancing innate and adaptive immune responses, particularly under chronic disease conditions.

## Objectives

We hypothesized that NLRP3 inflammasome-dependent factors induce the generation of MDSCs in MWS.

## Methods

We studied granulocytic MDSC numbers in 25 MWS patients under anti-IL-1 therapy with canakinumab and 20 healthy controls. After Ficoll density gradient sedimentation, granulocytic MDSCs were characterized as CD33^high^CD66b^high^IL-4Ra^inter^HLA-DR^low ^neutrophilic cells in the PBMC fraction, according to previously established human MDSC analysis methods. The functionality of MACS-isolated MDSCs was assessed using polyclonal T cell proliferation and cytokine/chemokine secretion tests. Physician's global assessment of disease activity, CRP, ESR, and T helper cell subsets were determined at the same time points and correlated with MDSC levels. Serum samples of 22 MWS patients and 5 healthy controls were examined by multiplex technique for possible MDSC inducing factors.

## Results

MWS patients under anti-IL-1 therapy displayed significantly elevated MDSC numbers (mean 1.65 ± 0.33%; range 0.16 - 5.17%) compared to healthy controls (mean 0.45 ± 0.05%; range 0.12 - 1.04%; *p *= 0.0025), although clinical MWS-disease activity was generally low at time of examination. MDSCs were functionally competent, as they suppressed polyclonal T cell proliferation, Th1, Th2, and Th17 responses. MDSCs correlated directly with Treg/Th17 and Treg/Th1 ratios indicating an influence on T helper cell subsets. Multiplex assays revealed the established MDSC-inducing growth factors GM-CSF and VEGF elevated in MWS sera even under anti-IL-1 therapy with canakinumab.

## Conclusion

MWS patients under anti-IL-1 therapy display significantly elevated numbers of granulocytic MDSCs. Increased MDSCs in MWS might represent a novel autologous anti-inflammatory mechanism in autoinflammatory conditions and may serve as a future therapeutic target.

## Disclosure of interest

N. Rieber Grant/Research Support from: NR, JKD, and DH obtained research grants from Novartis GmbH, Consultant for: NR and JKD took part in advisory boards for Novartis GmbH, A. Brand: None Declared, D. Neri: None Declared, T. Hall: None Declared, I. Schäfer: None Declared, S. Hansmann: None Declared, J. Kümmerle-Deschner: None Declared, D. Hartl: None Declared.

